# Economic cost-benefit analysis of person-centred medicines reviews by general practice pharmacists

**DOI:** 10.1007/s11096-024-01732-y

**Published:** 2024-05-30

**Authors:** Cian O’Mahony, Kieran Dalton, Leon O’Hagan, Kevin D. Murphy, Clare Kinahan, Emma Coyle, Laura J. Sahm, Stephen Byrne, Ciara Kirke

**Affiliations:** 1 Pharmaceutical Care Research Group, School of Pharmacy, University College Cork, Cork, Ireland; 2 Primary Care, Community Healthcare Organisations 1 and 8, Health Service Executive, Dublin, Ireland; 3 National Quality and Patient Safety Directorate, Health Service Executive, Dublin, Ireland

**Keywords:** Cost, General practice, Pharmacist, Pharmacoeconomics, Polypharmacy

## Abstract

**Background:**

Medicines reviews by general practice pharmacists improve patient outcomes, but little is known about the associated economic outcomes, particularly in patients at higher risk of medicines-related harm.

**Aim:**

To conduct an economic cost-benefit analysis of pharmacists providing person-centred medicines reviews to patients with hyperpolypharmacy (prescribed ≥ 10 regular medicines) and/or at high risk of medicines-related harm across multiple general practice settings.

**Method:**

Service delivery costs were calculated based on the pharmacist’s salary, recorded timings, and a general practitioner fee. Direct cost savings were calculated from the cost change of patients’ medicines post review, projected over 1 year. Indirect savings were calculated using two models, a population-based model for avoidance of hospital admissions due to adverse drug reactions and an intervention-based model applying a probability of adverse drug reaction avoidance. Sensitivity analyses were performed using varying workday scenarios.

**Results:**

Based on 1471 patients (88.3% with hyperpolypharmacy), the cost of service delivery was €153 per review. Using the population-based model, net cost savings ranging from €198 to €288 per patient review and from €73,317 to €177,696 per annum per pharmacist were calculated. Using the intervention-based model, net cost savings of €651–€741 per review, with corresponding annual savings of €240,870–€457,197 per annum per pharmacist, were calculated. Savings ratios ranged from 181 to 584% across all models and inputs.

**Conclusion:**

Person-centred medicines reviews by general practice pharmacists for patients at high risk of medicines-related harm result in substantial cost savings. Wider investment in general practice pharmacists will be beneficial to minimise both patient harm and healthcare system expenditure.

**Supplementary Information:**

The online version contains supplementary material available at 10.1007/s11096-024-01732-y.

## Impact statements

- Integration of pharmacists into general practice to deliver structured person-centred medicines reviews is economically dominant, delivering a return on investment far above expenditure on service delivery.
- General practice pharmacist-led medicines reviews produce significant savings by decreasing medicines costs and preventing drug-related hospital admissions.
- Investment in general practice pharmacists should be considered more widely due to the patient safety benefits alongside the economic benefits to health systems.

## Introduction

The prevalence of polypharmacy, most commonly defined as the use of ≥5 medicines concomitantly [1], is common and has risen significantly in recent decades. In Ireland, 60.4% of people aged ≥65 years had polypharmacy in 2012, an increase from 17.8% in 1997 [2]. Whilst polypharmacy can be appropriate and often necessary to manage multiple conditions [3], it is associated with a range of negative health outcomes, including adverse drug reactions (ADRs), reduced quality of life, hospitalisation, frailty, and overall mortality [4–7].

Potentially inappropriate prescribing (PIP) occurs where the risks of prescribing or not prescribing a medicine outweigh the potential benefits in a particular patient [8]. PIP is strongly associated with polypharmacy [9, 10] and similar negative effects for patients just outlined [11–13], as well as augmenting costs to healthcare systems. A high prevalence of PIP has been identified in Ireland, with a total expenditure on PIP in those aged ≥70 years of €45 million in 2007 [14]. Whilst the increase in medicines usage drives direct costs [15], the negative clinical outcomes associated with these phenomena result in potentially significant indirect costs due to increased healthcare utilisation—including doctor or emergency department visits, hospital admissions, and bed days [7, 11, 14, 16].

Interventions to optimise medicines use have shown unclear benefit overall [17]; however, pharmacist reviews of medicines have been shown to provide certain significant clinical and cost-saving benefits. These include reducing ADR risk [18–20], improving clinical parameters (e.g. blood pressure, glycosylated haemoglobin, cholesterol) [18, 19, 21], enhancing medicines adherence [20, 22], increasing medicines appropriateness [20], decreasing the total number of medicines taken [23–25], as well as reducing healthcare utilisation (e.g. doctor visits, hospital admissions) [20]. Furthermore, interdisciplinary collaboration of doctors and pharmacists [26], alongside patient involvement [27], has been shown to achieve enhanced safety and efficacy in medicines use. In primary care, the integration of pharmacists into general practice settings has shown success in reducing PIP and the number of medicines, and may decrease GP workload and emergency department attendance [26, 28].

Whilst pharmacist integration into these settings appears to be cost-effective [29], the evidence that interventions to optimise medicines use may provide benefits that outweigh their implementation costs remains limited [30]. This is further complicated by the limited evidence of the concrete clinical outcomes of medicines reviews, such as quality of life and mortality [31]. With that said, given the rising prevalence of polypharmacy and the associated increase in medicines complexity and risk of drug-related hospitalisations, there is a clear need to demonstrate greater evidence of the cost-effectiveness of pharmacist-led medicines reviews in general practice settings, particularly in patients at high risk of ADRs [28].

### Aim

This economic evaluation aimed to conduct an economic cost-benefit analysis of a pharmacist-led medicines review service across multiple Irish general practice settings involving patients with hyperpolypharmacy (prescribed ≥10 regular medicines) and/or at high risk of medicines-related harm, by:

1. calculating the cost of applying the intervention, and
2. assessing cost savings associated with changes in medicines usage post review and the prevention of potential ADRs and associated healthcare costs, by using:
  (i) a population-based model for avoidance of ADR-related hospital admission.
  (ii) a model based on ADR avoidance through interventions post medicines review.

### Ethics approval

Advice was sought from Health Service Executive (HSE) Dublin North East Research Ethics Committee and the Irish College of General Practitioners Ethics Committees. A full ethics submission and approval was not advised in the context of the work meeting the criteria of a service evaluation under guidance [32] from the HSE (Ireland’s national health services provider). The project complied with full information governance and data protection requirements, including patient consent for data collection, processing, and analysis.

## Method

### Guidelines

This paper was reported in accordance with the Consolidated Health Economic Evaluation Reporting Standards (CHEERS) guidelines for reporting health economic evaluations [33].

### iSIMPATHY project and patient eligibility

This paper reports on medicines reviews conducted by pharmacists integrated into general practices in Ireland as part of the European Union-funded **i**mplementing **S**timulating **I**nnovation in the **M**anagement of **P**olypharmacy and **A**dherence **Th**rough the **Y**ears (iSIMPATHY) project. iSIMPATHY aimed to improve the health and well-being of people at higher risk of medicines-related harm through delivery of medicines reviews in Scotland, Northern Ireland, and Ireland [34].

General practices in border counties in Ireland (i.e. counties near the border of Northern Ireland) expressing interest in participation were selected (*n* = 10) to ensure a population of approximately 20,000 patients for each pharmacist. To be eligible for iSIMPATHY review, patients originally had to meet ≥1 of the following criteria:

1. Prescribed ≥10 regular medicines.
2. At greater risk of adverse outcomes due to the prescription of ≥1 higher risk medicine or combinations of medicines or due to the underprescribing of potentially beneficial medicines [35].
3. Adults of any age with a possible shortened life expectancy (Appendix 1) [3].
4. Aged ≥50 years and living in a residential care setting.

The iSIMPATHY project later broadened the inclusion criteria to increase patient recruitment, which meant that from October 2021 onwards in Ireland: criterion 1 above was changed to ‘*prescribed* ≥*5 regular medicines*’ and criterion 2 was expanded to also include patients on a high-risk medicine or combination of medicines as per the pharmacist's clinical judgement. However, only patients meeting the original criteria 1–4 above who participated in a medicines review from January 2021 to December 2022 (i.e. the iSIMPATHY data collection period), and who consented to data collection and analysis were included in this economic evaluation, as the aim was to evaluate the interventions performed in a more homogenous group of higher priority patients (i.e. those with hyperpolypharmacy and/or at a higher risk of medicines-related harm).

### iSIMPATHY medicines reviews

Pharmacists prepared for, and then conducted, a structured person-centred medicines review with a patient using the ‘*7 steps to appropriate polypharmacy*’ approach [34]. Interviews were conducted via telephone or face to face, depending on patient preference and restrictions associated with the COVID-19 pandemic. Reviews sought to improve patients’ medicines understanding and adherence, with medicines optimisation through shared decision-making. The pharmacist communicated a review summary and recommendations to the general practitioner (GP) and the GP and pharmacist actioned changes as appropriate. The pharmacist also contacted other healthcare professionals including specialist teams and community pharmacists as necessary. Follow-up with patients and healthcare professionals typically occurred 2–6 weeks post review, but varied from 0 to 12 weeks depending on intervention complexity and/or the time required to assess resolution. Each “*review*” related to a unique patient.

### Cost evaluation

This is a retrospective analysis that combines pre-planned analyses from methodology outlined in the original iSIMPATHY project [36] and multiple post-hoc analyses to give greater confidence to the estimates. The healthcare provider perspective was taken for this cost evaluation. All costs reported are in Euro (€). Where cost values from other currencies were used from literature, these were converted to Euro using 2022 Purchase Parity Power (PPP), as recommended [37, 38]. Costs for the year 2022 were applied to all data. Given that all data were collected over a 2-year period, no discounting effects were applied to cost figures.

### Costs

The unit cost for pharmacist time was calculated from the midpoint of the senior pharmacist pay scale [39], pension payments, social insurance, and overheads—in accordance with guidelines for economic evaluations in Ireland [40]. Overheads costs were adjusted based on an audit conducted within the study. Pharmacists recorded the time to prepare for, carry out, and follow up after each review. Pharmacist cost was calculated by multiplying the unit pharmacist cost per minute by the mean total review time. Two sensitivity analyses were performed:

1. Pharmacists allow one workday per week for non-review activities (meetings etc.).
2. The actual number of reviews conducted annually within the iSIMPATHY project, allowing for data collection and project-related activities (approximating two workdays per week).

The GP fee per review was a constant of €17.50; this was based on a previously reported rate for a similar service in Ireland [41].

### Savings

Direct cost savings driven by changes in medicines usage were calculated. Exact cost consequences of the medicines review were calculated for a subset of 40 patients, with a random sample of 10 completed reviews per pharmacist selected. The cost of medicines changed (inclusive of wholesaler drug price and rebate where applicable, pharmacy fees, and value added tax where applicable) as a result of the review were calculated with reference to the Primary Care Reimbursement Scheme drug file for March 2022 [42], in line with national guidance [43], and extrapolated to calculate the annual direct cost avoidance. Bootstrapping generated 1000 samples for sample estimates calculation.

Two methods were used to determine indirect cost savings:

1. Population-based avoidance of ADR-related admission method: population rates of hospital admissions and the likelihood that an emergency admission was associated with an ADR [44] were used to calculate the likelihood an individual would be admitted to hospital with an ADR annually. An assumption was made that 25% of these ADR admissions would be avoided if that person received an iSIMPATHY medicines review. Whilst a meta-analysis has found that pharmacist-led interventions in older adults reduce the risk of any ADR by 35% [45], the iSIMPATHY report (across three countries) found that 26% fewer patients reported experiencing side effects from their medicines post review (38%) versus pre review—informing the 25% assumption [34]. Cost avoidance was calculated by multiplying the number of hospital ADR admissions potentially avoided by healthcare costs associated with such an admission, including follow-up costs post hospitalisation [46].
2. Intervention-based avoidance of ADR method: upon medicines review completion, any changes that were made to a patient’s medicines were evaluated using the six-point Eadon scale to grade the potential clinical significance of interventions [47]. All study pharmacists were trained to provide a standardised grading of intervention significance (including a quality assurance process). This was to be completed for a minimum of 50% of the patients, and the initial target was exceeded.

The Eadon scale was mapped to methodology set out by Nesbit et al. [48], assigning a score of the probability of an ADR occurring, as seen in Table 1. To prevent multiple interventions in each patient from stacking probability-wise, only the highest probability (i.e., highest Eadon grade) intervention for each patient was used to calculate potential cost avoidance.

**Table 1:** Mapping Nesbit ADR avoidance to Eadon grading

Probability of ADR occurring (Nesbit)	Probability score (Nesbit)	Eadon grade*
No harm expected	0	2: Intervention is of no significance to patient care
Very Low	0.01	3: Intervention is significant but does not lead to an improvement in patient care
Low	0.1	4: Intervention is significant and results in an improvement in the standard of care
Medium	0.4	5: Intervention is very significant and prevents a major organ failure or adverse reaction of similar importance
High	0.6	6: Intervention is potentially life saving

*No Eadon grades of 1 (“Intervention is detrimental to patient’s well-being”) were recorded, hence this was excluded

Cost avoidance was calculated from the probability of the review averting an ADR (Table 1) and the unit cost for an avoided ADR in an ambulatory care setting [49]. All data were analysed using Microsoft® Excel.

## Results

**Fig. 1:**
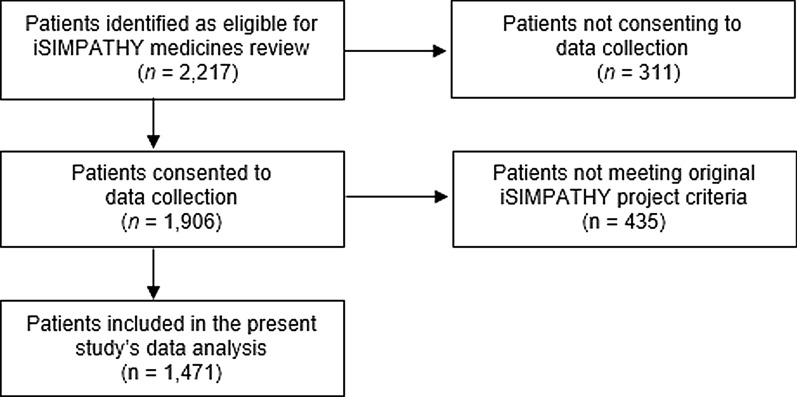
Flow diagram showing recruitment and eligibility process

Of the 2,217 iSIMPATHY patients reviewed in Ireland, 1906 (86%) agreed to data collection. Data relating to 1471 patients were analysed for this economic evaluation. Of these, 1299 (88.3%) had hyperpolypharmacy at the time of review; the remaining 172 were prescribed < 10 medicines but had ≥1 risk factor as per the inclusion criteria. Figure 1 details the patient eligibility process. The mean age was 76.0 years (standard deviation [SD] ± 9.5), with 90.1% of patients aged ≥65 years, whilst the mean number of comorbidities was 6.2 (SD ± 2.3) and the mean number of medicines was 13.8 (SD ± 4.7) before the review. A total of 125 patients (8.5%) were deemed as having a shortened life expectancy (Appendix 1).

**Fig. 2:**
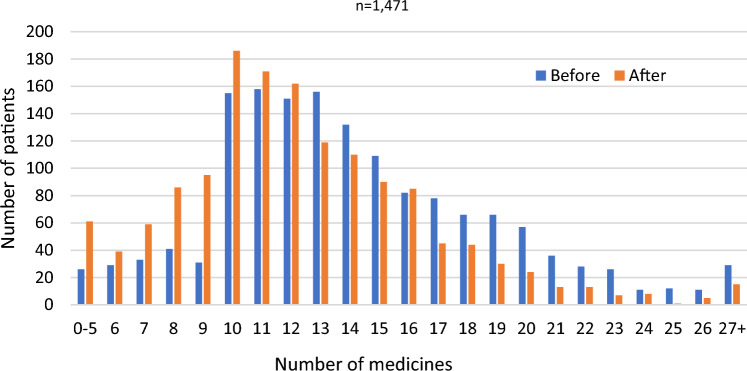
Number of medicines before and after review (n = 1471 patients)

A mean of 12.0 (SD ± 4.2) interventions were made and a mean reduction of 1.5 (SD ± 2.0) medicines achieved per patient review. The distribution of total number of medicines is illustrated in Fig. 2.

### Cost analysis

The total pharmacist annual cost was calculated to be €83,482. The mean total pharmacist time was 157 min (SD ± 46) per patient reviewed. Adjusting for leave and national holidays, a pharmacist would work 218 days per year or 96,792 min per year. This gives a pharmacist cost of €135.50 per review. Given the GP payment of €17.50 per review, this gives a cost per review of €153. After adjusting for leave allowances, pharmacists complete 218 working days each year. If all of the pharmacist’s time was available for review-related activity, 617 reviews would be delivered annually at a cost of €94,271 per annum.

The mean reduction in drug costs per review was found to provide an annual cost saving of €376 (95% confidence interval €212–€540) per patient reviewed. Applying this unit cost to the 617 annual reviews gives a total direct cost saving of €231,992 per annum.

### Population-based savings

The cost of an ADR-related hospitalisation and follow-up healthcare was €9927 [45]. Assuming 25% of ADR-related hospitalisations at population level are avoidable through medicines review, an indirect cost saving of €65 per review was observed. Total cost savings were therefore determined to be €441 per review. This results in net cost savings of €288 per review, or €177,696 per annum per pharmacist.

Using the cost savings per review (€441) as a constant and adjusting for the total amount of working days per year, sensitivity analyses determined that pharmacists conducting review activity 4 days per week would achieve net cost savings of €124,968 per annum, or €254 per review per pharmacist. Review activity was conducted approximately 3 days per week during the iSIMPATHY project, due to project requirements, resulting in net cost savings of €73,317 per annum, or €198 per review per pharmacist.

### Intervention-based savings

Adjusted for inflation and PPP, the cost of an ADR in primary care was found to be €2548. Eadon gradings were available for 1238 patients. Table 2 describes the indirect cost avoidance of avoided ADRs as calculated by the Nesbit et al*.* methodology, which resulted in a cost saving of €518 per review.

**Table 2:** ADR cost avoidance using Nesbit et al. method

Probability of ADR occurring	Eadon grade	Number of interventions	Probability score	Number of patients*	Total cost savings (€)
No harm expected	2	230	0.00	0	0
Very Low	3	2,348	0.01	0	0
Low	4	11,552	0.1	811	206,643
Medium	5	582	0.4	427	435,198
High	6	0	0.6	0	0
Total		14,712		1238	641,841
Total per review					518

*Number of patients whose highest graded intervention occurred at this grade

Combining with the cost saving per review including drug cost savings (€376), total cost savings with this method were €894 per review. This results in net cost savings of €741 per review, with corresponding annual savings of €457,197. Sensitivity analyses showed annual cost savings of €347,844 allowing for one non-review day per week and annual cost savings of €240,870 based on actual total study outputs.

A savings ratio of 288% is achieved with the conservative population-based model and 584% with the intervention-based model when allowing for 5 review days per week. The findings indicate that this service is net cost-saving, with reduced drug costs post review alone providing a substantial savings ratio of 246% (or €137,591 per annum).

The study findings are summarised in Table 3, where all figures are costs per review unless specifically stated.

**Table 3:** Summary of findings

Model	Review days (Corresponding annual reviews)	Costs (€)	Cost savings (€)	Net cost savings (€)	Net annual cost savings (€)	Savings ratio (%)
Population-based	5 days per week (617)	153	441	288	177,696	288
	4 days per week (492)	187	441	254	124,968	236
	3 days per week (370)	243	441	198	73,317	181
Intervention-based	5 days per week (617)	153	894	741	457,197	584
	4 days per week (492)	187	894	707	347,844	478
	3 days per week (370)	243	894	651	240,870	368

## Discussion

### Statement of key findings

This collaborative, structured person-centred medicines review service by pharmacists working in multiple general practices was effective, delivering a large number of reviews with benefits that included reducing the number of medicines, the risk of medicines-related harm, and additional healthcare utilisation. Net cost savings of up to €450,000 per annum may be achieved. This service is economically dominant under all modelling and sensitivity analyses performed.

This was a medicines optimisation intervention, with a holistic approach to reducing, increasing, or changing doses of patients’ medicines through building shared understanding and decision-making with the patient. Even with medicines additions where applicable, a substantial reduction in drug costs was achieved overall, with annual savings of over €200,000 calculated per pharmacist.

### Strengths and weaknesses

The present study provides a robust analysis of 1471 medicine reviews of patients at high risk of medicines-related harm by four pharmacists working across 10 general practices over a 2-year period. National hospitalisation rate data is only available for all ages or those ≥65 years of age. Given that > 90% of the sample were aged ≥65 years, the second was chosen as being more representative of the sample. For patients with a shortened life expectancy (8.5% of total), an assumption of 12-month survival was made due to unavailability of survival data; it is difficult to predict the exact effect of this assumption on costs, e.g. given the potential for either reduced medication costs versus increased healthcare utilisation. A subset of 40 randomly selected patients was used to calculate direct cost savings by a reduction in medicines use pre and post review. The figure of €376 per review is quite similar to a gross calculation of medicines cost savings calculated by the average number of medicines reduced (1.5) multiplied by the average reimbursable medicines cost (€215), which comes to €323 per review [42]. This adds confidence to the accuracy of the drug cost analysis. The increased accuracy of an exact cost calculation for changes in medicines post review is a further strength. The time recordings in this study were self-reported, which may lead to bias. There may be a slight under-recording of review time, given that some queries may have emerged (and were potentially resolved) after data collection was completed. Furthermore, this study though did not account for all the possible economic costs and benefits (e.g. patients’ increased health status and other societal impacts), so this scope must be accounted for when interpreting the findings.

The Eadon [47] and Nesbit [48] grading tools had not previously been mapped as in the present study. Given the similarities of clinical description at each stage of the scales, the two tools seem suited for co-use. To prevent multiple interventions in each patient from stacking probability-wise, only the highest probability (i.e., highest Eadon score) intervention was used to calculate potential cost avoidance.

### Interpretation and implications for future research and practice

Pharmacist-led medicines reviews in primary care settings have consistently demonstrated positive effects on clinical markers [20, 21], reductions in the number of medicines and enhanced medicines appropriateness [20], improved adherence [20], and decreased association with hospitalisation [21]—all of which are likely to translate to substantial cost savings. However, research evaluating the economic impact of pharmacist-led medicines reviews shows mixed findings from limited studies [50, 51]. A heterogeneity of medicines review types and settings have been reported on, with various methods of cost calculation employed. Notably though, studies including similar models to ours have demonstrated similar direct savings [52], and therefore these models should be considered for evaluations like this in future.

The relative impact of the cost savings provided by potentially preventing an ADR-related hospital admission, calculated on a population ADR admission rate basis, was low. An assumption that a pharmacist review would prevent 25% of the potential ADR admissions was used in this analysis. Although reviews were not confined to older adults, the inclusion criteria and factors relating to searches and services in the practices led to a primarily older population participating in reviews, with 90.1% of patients reviewed being aged ≥65 years. The economic analysis has thus based calculations for indirect savings on frequency and costs of hospital admissions for adults aged ≥65 years. The assumption of 25% could be lowered further with minimal impact on cost savings. This model using population data for ADR avoidance could be said to be the lower limit for the cost savings provided by this service, given that the data are based on the average older adult ADR-related hospitalisation rate. Given the high-risk nature of the patients engaging in these medicines reviews, more ADR admissions would be anticipated and therefore preventable through patient review [6], meaning the €288 net saving per review is likely to be an underestimate. Similarly, the €742 net savings per review calculated using the intervention-based model are also likely to understate these benefits. This model assigned a likelihood of avoided ADRs for one intervention per patient, whereas the mean was 12 interventions per patient—with the majority (79.2%) adjudicated to have significantly improved patient care, thus improving the possibility of greater cost savings.

This study provides clear evidence of the economic benefits of pharmacist-delivered person-centred reviews in general practices in Ireland. While pharmacists have been successfully integrated into this setting in some countries [18, 50, 53, 54], there has been little to no integration of any significance in Ireland, as with many other jurisdictions. Therefore, this study provides economic justification for implementing on a wider scale in Ireland and piloting in other countries. Given that this study specifically evaluated the cost and benefits in patients with hyperpolypharmacy and/or at high risk of medicines-related harm, this group are possibly those most likely to benefit from any upscaling of general practice pharmacist medicines reviews and the economic benefits are greatest in this group.

The present study evaluated general practices who voluntarily participated in the project, which may indicate greater acceptance to pharmacist integration and an interdisciplinary approach. Recent research has highlighted that both GPs and pharmacists not personally exposed previously to such pharmacist roles were open to pharmacist integration; however, both groups identified concerns with funding such roles and perceived that government funding would be essential to widely establish the role [55–57]. This study supports the economic case for public funding of this service, with a substantial return on investment demonstrated.

## Conclusion

This economic evaluation has demonstrated that pharmacist-led person-centred medicines reviews can be delivered across multiple general practice settings and result in substantial cost savings from the healthcare provider perspective. These pharmacist-led medicines reviews helped reduce the medicines burden for patients, as well as minimising medicines-related harm. Investment in integration of pharmacists into general practices to deliver this service will result in improved patient outcomes alongside the reduction of costs associated with medicines and the management of ADRs and drug-related hospitalisations.

## Supplementary Information

Below is the link to the electronic supplementary material.

Supplementary file1 (DOCX 20 kb)
