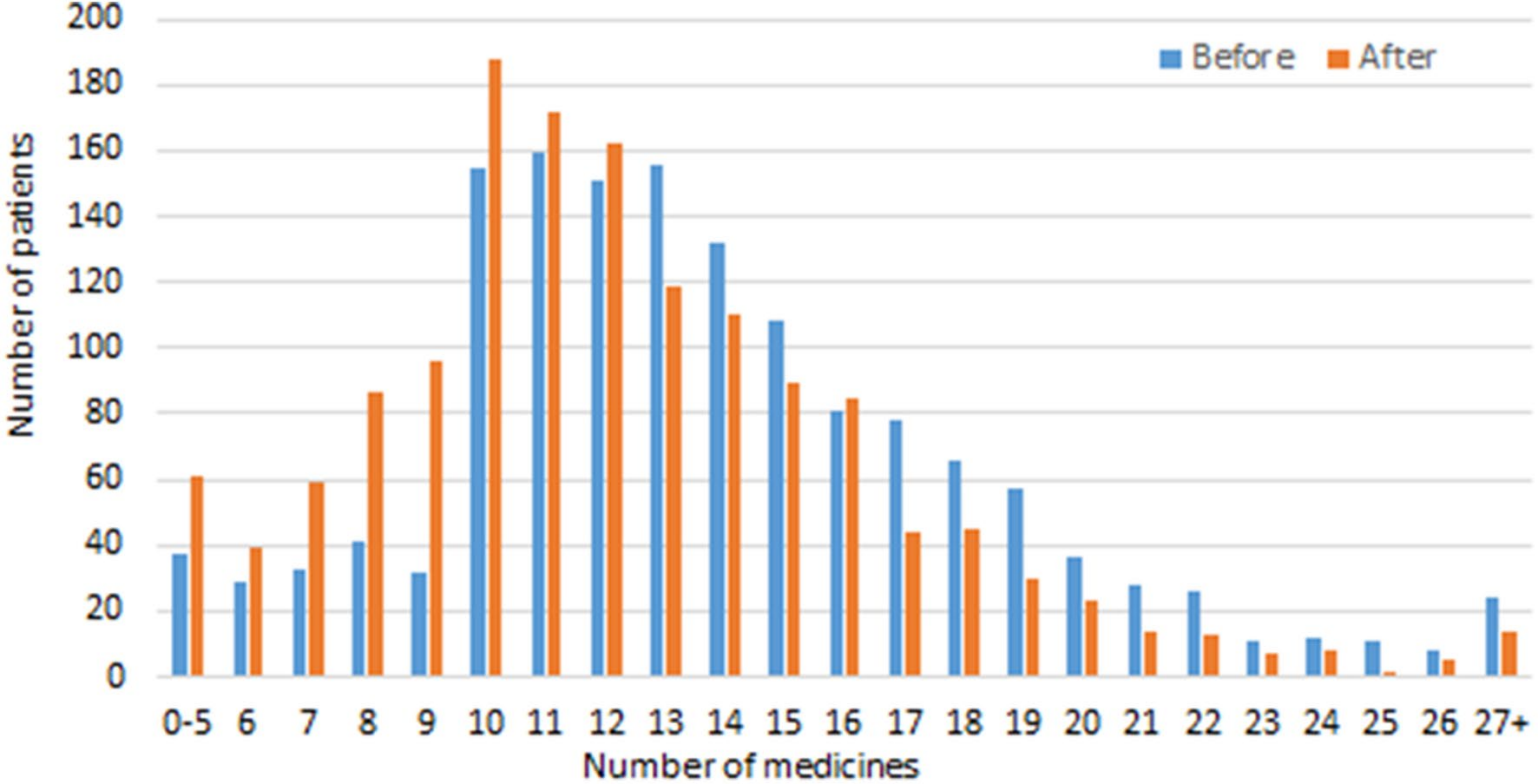# Correction: Economic cost-benefit analysis of person-centred medicines reviews by general practice pharmacists

**DOI:** 10.1007/s11096-026-02116-0

**Published:** 2026-05-05

**Authors:** Cian O’Mahony, Kieran Dalton, Leon O’Hagan, Kevin D. Murphy, Clare Kinahan, Emma Coyle, Laura J. Sahm, Stephen Byrne, Ciara Kirke

**Affiliations:** 1 Pharmaceutical Care Research Group, School of Pharmacy, University College Cork, Cork, Ireland; 2 Primary Care, Community Healthcare Organisations 1 and 8, Health Service Executive, Dublin, Ireland; 3 National Quality and Patient Safety Directorate, Health Service Executive, Dublin, Ireland

**Correction to: International Journal of Clinical Pharmacy (2024) 46:957–965** 10.1007/s11096-024-01732-y

In the original publication, several values reported in the *Results* section and *Discussion *section require corrections:

- The standard deviation of age was reported as **9.7**; the correct value is **9.5**.
- The mean number of comorbidities was reported as **6.1**; the correct value is **6.2**.
- The number of patients prescribed 10 or more medicines (hyperpolypharmacy) was reported as **1301 (88.4%)**; the correct value is **1299 (88.3%)**.
- The mean reduction in the number of medicines was reported as **1.6 (SD 2.1)**; the correct value is **1.5 (SD 2.0)**.
- In the Discussion section, the average number of medicines reduced value has been corrected from 1.6 to 1.5, and €344 to €323. The correct sentence should read as “…the average number of medicines reduced (1.5) multiplied by the average reimbursable medicines cost (€215), which comes to €323 per review”

The result section should read as follows:

Of the 2,217 iSIMPATHY patients reviewed in Ireland, 1906 (86%) agreed to data collection. Data relating to 1471 patients were analysed for this economic evaluation. Of these, 1299 (88.3%) had hyperpolypharmacy at the time of review; the remaining 172 were prescribed < 10 medicines but had ≥ 1 risk factor as per the inclusion criteria. Figure 1 details the patient eligibility process. The mean age was 76.0 years (standard deviation [SD] ± 9.5), with 90.1% of patients aged ≥ 65 years, whilst the mean number of comorbidities was 6.2 (SD ± 2.3) and the mean number of medicines was 13.8 (SD ± 4.7) before the review. A total of 125 patients (8.5%) were deemed as having a shortened life expectancy (Appendix 1).

A mean of 12.0 (SD ± 4.2) interventions were made and a mean reduction of 1.5 (SD ± 2.0) medicines achieved per patient review. The distribution of total number of medicines is illustrated in Fig. 2.

The Results subsection in the abstract has also been updated as follows “Based on 1471 patients (88.3% with hyperpolypharmacy), the cost of service delivery was €153 per review. Using the population-based model, net cost savings ranging from €198 to €288 per patient review and from €73,317 to €177,696 per annum per pharmacist were calculated. Using the intervention-based model, net cost savings of €651–€741 per review, with corresponding annual savings of €240,870–€457,197 per annum per pharmacist, were calculated. Savings ratios ranged from 181 to 584% across all models and inputs.”

Additionally, Figure 2 has been revised to reflect the changes affecting the numbers of medicines. For completeness and transparency, the old incorrect version and the corrected version of Fig. 2 are displayed below.

Incorrect Figure 2:

Fig. 2 Number of medicines before and after review (n = 1471 patients).
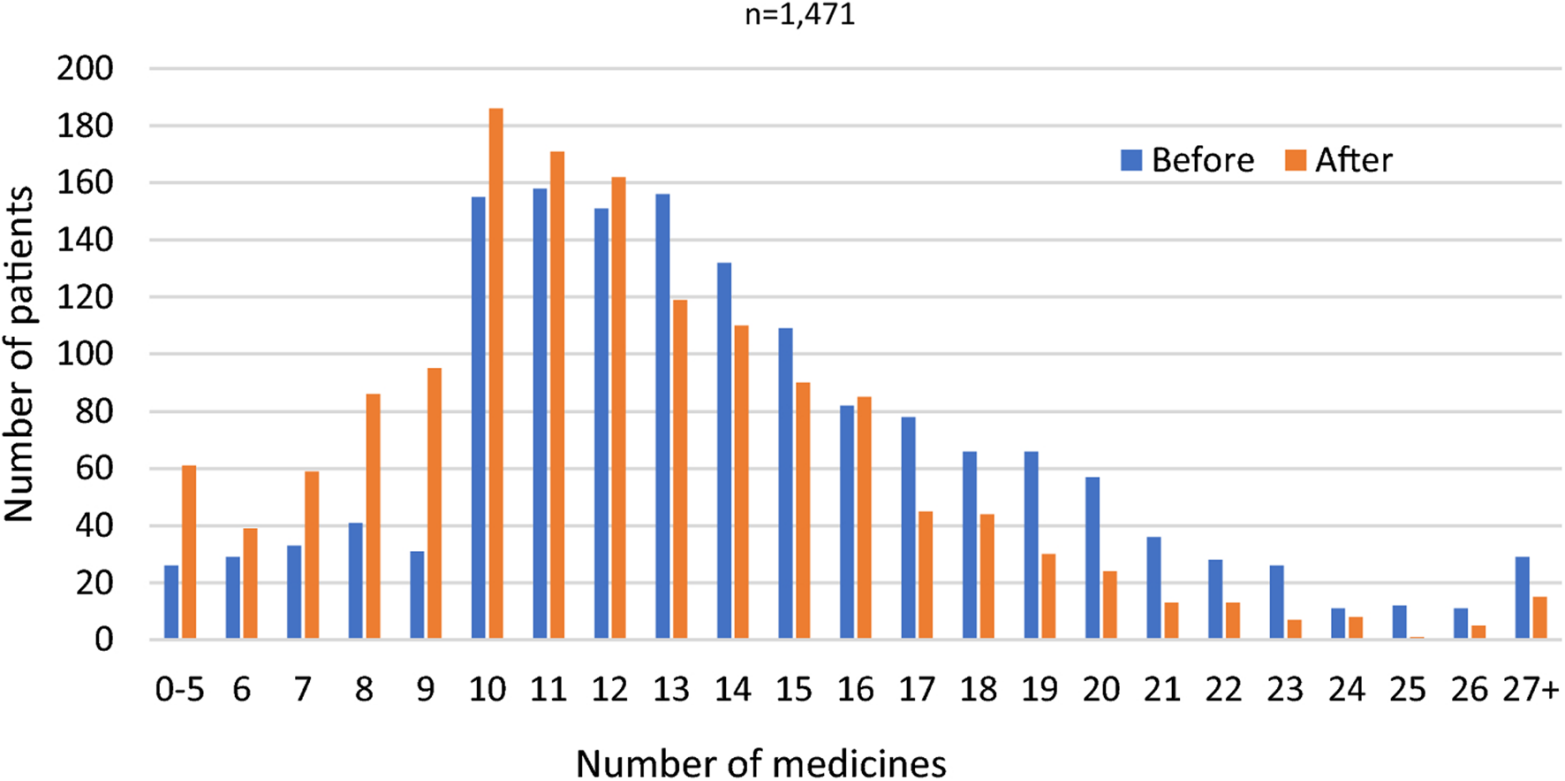


Correct Figure 2:

Fig. 2 Number of medicines before and after review (n = 1471 patients).