# Supplementary material for: Economic cost-benefit analysis of person-centred medicines reviews by general practice pharmacists
Source: Int J Clin Pharm. 2024 May 30;46(4):957–65. doi: 10.1007/s11096-024-01732-y (PMC11286700; doi:10.1007/s11096-024-01732-y)
Supplement: Supplementary file 1 — Supplementary file1 (DOCX 20 kb) [file 11096_2024_1732_MOESM1_ESM.docx]

**Appendix 1****: Criteria for shortened life expectancy**

Patients meeting the criterion of ‘*shortened life expectancy*’ would have to have met ≥1 criterion^1^:

- Where ‘no’ is the answer to the question, ‘*would you be surprised if this person were to die in the next 6 to 12 months?*’
- Where a patient with advanced disease is making a choice for comfort care rather than curative treatment.
- Where help is required for multiple activities of daily living, either at home or in care home due to:
  - advanced organ failure
  - multiple comorbidity giving significant impairment in day-to-day function
  - advanced dementia.

^1^ Scottish Government Polypharmacy Model of Care Group. Polypharmacy Guidance, Realistic Prescribing 3rd Edition, 2018. Scottish Government.
